# Kawasaki disease and subsequent risk of allergic diseases: a population-based matched cohort study

**DOI:** 10.1186/1471-2431-13-38

**Published:** 2013-03-23

**Authors:** Ho-Chang Kuo, Wei-Chiao Chang, Kuender D Yang, Hong-Ren Yu, Chih-Lu Wang, Shu-Chen Ho, Chun-Yuh Yang

**Affiliations:** 1 Department of Pediatrics, Kaohsiung Chang Gung Memorial Hospital and Chang Gung University College of Medicine, Kaohsiung, Taiwan; 2 Department of Clinical Pharmacy; Master Program for Clinical Pharmacogenomics and Pharmacoproteomics, School of Pharmacy, Taipei Medical University, Taipei, Taiwan; 3 The Department of Medical Research and Development of Pediatrics, Show Chwan Memorial Hospital in Chang Bing, Changhua, Taiwan; 4 Institute of Clinical Medical Sciences, National Yang Ming University, Taipei, Taiwan; 5 Department of Pediatrics, Po-Jen Hospital, Kaohsiung, Taiwan; 6 Department of Public Health, College of Health Sciences, Kaohsiung Medical University, Kaohsiung, Taiwan

**Keywords:** Kawasaki disease, Allergic disease, Cohort study

## Abstract

**Background:**

The risk of allergic diseases among Kawasaki disease (KD) patients relative to the general population is not known. The aim of this study was to perform a population-based cohort study to investigate the risk of allergic diseases among children after KD in Taiwan- a country with the third highest incidence of KD in the world.

**Methods:**

Data were obtained from the Taiwan National Health Insurance Research Database. In total, 253 patients who were 5 years of age or younger and had a first-time hospitalization with a diagnosis of KD between 1997 and 2005 were included as the study cohort and 1,012 non-KD patients matched for age and sex were included as comparison cohort. Multivariate Cox proportional hazard regression model was used to adjust for confounding and to compare the 6-year allergic-free survival rate between these two cohorts.

**Results:**

The incidence rate of allergic diseases (184.66 per 1000 person-year) was significantly higher in the KD cohort than in the control cohort (124.99 per 1000 person-years). After adjusting for potential confounders, the adjusted hazard ratios of asthma and allergic rhinitis were 1.51 (95% confidence interval = 1.17-1.95) and 1.30 (95% confidence interval = 1.04-1.62), respectively.

**Conclusion:**

We conclude that KD patients were at an increased risk for allergic diseases compared with the comparison cohort.

## Background

Kawasaki disease (KD) is an acute febrile systemic vasculitis that was first described by Kawasaki et al. in 1974 [1]. In developed countries, it is the leading cause of acquired heart disease in children but its etiology remains unknown [2-4]. This acute illness presents with systemic inflammation and occurs with fever lasting at least 5 days, with conjunctival and oral mucosa changes, fissured lips, cervical lymphadenopathy, skin rash, and palm/sole erythema/induration [5,6]. The most serious complication of KD is the presence of coronary artery lesions (CAL), including myocardial infarction, coronary artery fistula formation [7], coronary artery dilatation, and coronary artery aneurysm (CAA) [8,9]. Children aged less than 5 years of age are the most susceptible population. According to recent epidemiologic studies, Asian populations have a much higher incidence of KD. Japan has the highest annual incidence in the world [10], followed by Korea and Taiwan (218, 113 and 69 per 100,000 children aged < 5 years of age, respectively) [11,12]. The incidence of KD has increased globally in recent years [9,10,13-15].

It has been reported that the prevalence of atopic dermatitis among children with KD was 9 times greater than that of controls [16]. A Japanese cross-sectional survey reported that atopic dermatitis and/or allergic rhinitis were more common in KD-affected children when compared to the non-KD controls [17]. A cross-sectional sibling study conducted in Singapore reported that KD-affected children had more allergy and asthma than their non-KD affected siblings [18]. These cross-sectional data suggest that KD tended to be associated with allergic diseases [17,18]. Recently, a case–control study found that KD patients were more likely to have been admitted to hospital for asthma/allergy than non-KD controls (OR = 2.6, 95% CI = 1.7-4.2) [19]. These data suggest that these allergic diseases are unlikely to reflect immune dysfunction resulting from KD itself [19].

To our knowledge, despite several reports of conditions associated with alergic diseases in KD patients (cross-sectional or case–control designs), large sample data regarding the exact incidence of allergic diseases occurring in KD patients are still lacking. We undertook the present study to determine the incidence of allergic diseases in KD patients in Taiwan.

## Methods

### Data source

The National Health Insurance (NHI) program, which provides compulsory universal health insurance, was implemented in Taiwan on March 1, 1995. Under the NHI, 98% of the island’s population receives all forms of health care services including outpatient services, inpatient care, Chinese medicine, dental care, childbirth, physical therapy, preventive health care, home care, and rehabilitation for chronic mental illness. In cooperation with the Bureau of NHI, the National Health Research Institute (NHRI) of Taiwan randomly sampled a representative database of 1,000,000 subjects from the entire NHI enrollees by means of a systematic sampling method for research purposes. There were no statistically significant differences in age, gender, and healthcare costs between the sample group and all enrollees, as reported by the NHRI. This dataset (from January 1996 to December 2010) includes all claim data for these 1,000,000 subjects, offers a good opportunity to examine the incidence of allergic diseases occurring among patients with KD. These databases have previously been used for epidemiological research, and information on prescription use, diagnoses, and hospitalizations has been shown to be of high quality [20].

Because the identification numbers of all individuals in the NHRI databases were encrypted to protect the privacy of the individuals, this study was exempt from full review by the Institution Review Board.

### Study cohorts

From this database, we selected all children who were 5 years of age or younger and had a first-time hospitalization with a diagnosis of KD (ICD-9-CM code 446.1) between 1997 and 2005 (n = 378). We excluded subjects who had been hospitalized with a KD before the year of 1997 (i.e., in 1996; because the NHI program started in 1995, we were able to trace the use of medical services only to 1996) to limit the study subjects to new cases (n = 31). We excluded subjects who had been given a diagnosis of allergic diseases including asthma (ICD-9 CM code 493.X) or allergic rhinitis (ICD-9 CM code 477.X) prior to their index ambulatory care visit (the date of a patient’s hospitalization for KD) (n = 93). In total, 254 patients with KD were included in the study group.

The comparison cohort was selected from the remaining patients in the database. We first excluded patients who had been diagnosed with KD during the period 1996–2010 and those older 5 years of age. Controls were also chosen from 1997 to 2005. For each study cohort patient, 4 reference individuals were identified randomly and matched for gender, age, and year of index ambulatory care visit. For comparison cohort, the index ambulatory care visit was their first ambulatory care visit occurring in the index year. Of the 254 incident KD cases ascertained, no controls could be found for one case. A total of 1,012 individuals served as a comparison cohort group.

### Potential confounders

For all individuals in the two cohorts, we obtained data on potential confounders including age, gender, number of physician visits 1 year before index date, the levels of insurance as an economic index and urbanization level of the community in which the patients resided. In Taiwan, the urbanization levels are divided into 7 categories, ranging from category 1 (the most urbanized) to category 7 (least urbanized), based on 5 indices: population density, percentages of residents with college or higher education, percentages of residents over 65 years old, percentage of residents who were agricultural workers, and the number of physicians per 100,000 people [21]. For the analyses, the urban–rural classification was aggregated into 3 levels: I. urban (categories 1 and 2), II. suburban (categories 3 and 4), and III. rural (categories 5, 6, and 7). All children were financially dependents of the insured (the ones who paid the insurance fee; i.e., usually the child’s parents, grandparents, or social welfare institutions). We used the monthly income of children’s enrollees as a proxy to measure of children’s socioeconomic status.

### Statistics

For comparisons of proportions between the study and comparison cohorts, chi-square statistics were used. We compared continuous data using the student t test. Each patient was individually tracked for a 5-year period starting from their cohort entry to identify whether the patient had experienced allergic rhinitis or asthma during the follow-up period. The person-years of follow-up were calculated for each patient from the date of the cohort entry to the date of being diagnosed with allergic diseases or the end of the study (a 5-year follow-up period), whichever occurred first. Incidence rates were calculated by dividing the number of allergic diseases by the total person-years of follow-up. Cox proportional hazards regression models were used to estimate the hazard ratios (HR) and 95% confidence intervals (CI) in a multivariate model adjusting for the above-mentioned potential confounders. The assumption of proportional hazards was assessed by including an interaction term between time and exposure (KD patients vs patients in the comparison cohort) in the model, and the proportional assumption was satisfied. Analyses were performed using the SAS statistical package (version 9.2, SAS Institute Inc., Cary, NC, USA). All statistical tests were two-sided. Values of p < 0.05 were considered statistically significant.

## Results

There were 253 patients in our KD cohort that were compared with 1,012 selected matched controls. Table 1 presents the distribution of demographic characteristics of the study subjects and controls. After matching for sex and age, patients with KD had comparable characteristics for monthly income of the insured and urbanicity of residence of the health insurance enrolled. However, patients with KD had more physician visit on average than patients in the comparison cohort (22.42 times vs 15.31 times).

**Table 1:** Basic characteristics for KD patients versus the comparison patients

**Variable**	**KD patients (n = 253)**		**Comparison patients (n = 1012)**		
	**n**	**%**	**n**	**%**	**p value**
Gender					
Male	152	60.08	608	60.08	-
Female	101	39.92	404	39.92	
Age (mean ± SD)	1.61 ± 1.20		1.61 ± 1.20		-
No. physician visits	22.42 ± 17.19		15.31 ± 12.82		< 0.001
0	10	3.95	83	8.20	< 0.001
1-8	50	19.76	297	29.35	
9-19	69	27.27	324	32.02	
≥ 20	124	49.01	308	30.43	
Urbanicity					
Urban	153	60.47	597	58.99	0.676
Suburban	80	31.63	339	33.49	
Rural	20	7.90	76	7.51	
Monthly income of children’s enrollee					
NT$ < 18300	74	29.25	322	31.82	0.189
NT$ 18301-25199	76	30.04	340	33.60	
NT$ ≥ 25200	103	40.71	350	34.52	

Table 2 shows the distribution of allergic diseases during the 5-year follow-up periods for these two cohorts. Of the total 1,265 sampled patients, 620 patients (49.01%) developed allergic diseases throughout the study periods, 148 (58.50% of the KD patients) from the study cohort (with an incidence rate of 184.66 per 1,000 person-years) and 472 (46.64%) from the comparison cohort (with an incidence rate of 124.99 per 1,000 person-years). The log-rank test showed that KD patients had significantly lower 5-year allergic disease-free survival rates than patients in the comparison cohort (p < 0.001).

**Table 2:** Rates and hazard ratios of allergic disease KD cohort compared to patients in comparison cohort during the 5-year follow-up period

	**Comparison patients (n = 1012)**	**KD patients (n = 253)**
Allergic disease		
Yes	472	148
Person time (years)	3776.39	801.46
Incidence^a^	124.99	184.66
Crude HR (95% CI)	1.00	1.57 (1.20-1.92)
Adjusted HR (95% CI)^b^	1.00	1.52 (1.24-1.86)
Asthma		
Yes	269	89
Person time (years)	4325.12	997.73
Incidence^a^	62.19	89.20
Crude HR (95% CI)	1.00	1.49 (1.16-1.92)
Adjusted HR (95% CI)^b^	1.00	1.51 (1.17-1.95)
Allergic rhinitis		
Yes	388	119
Person time (years)	4064.97	922.37
Incidence^a^	95.45	129.02
Crude HR (95% CI)	1.00	1.39 (1.12-1.72)
Adjusted HR (95% CI)^b^	1.00	1.30 (1.04-1.62)

^a^ per 1000 person-years.

^b^ Adjustments are made for age, gender**,** and number of physician visits.

Table 2 also shows the crude and adjusted hazard ratios (HR) for allergic diseases for the two groups. After adjusting for potential confounders, the HR for asthma for KD patients was 1.51 (95% CI = 1.17-1.95) compared to patients in the comparison cohort. KD patients were at a 1.30-fold risk for allergic rhinitis compared with patients in comparison cohort (HR = 1.30, 95% CI = 1.04-1.62).

## Discussion

To our knowledge, this is the first population-based cohort study to examine the relationship between KD and the subsequent risk of allergic diseases (asthma and allergic rhinitis). Previous studies used either cross-sectional [16-18] or case–control designs [19] to investigate the prevalence of asthma/allergic diseases in KD patients and non-KD controls.

Previous studies reported that the majority of the increased asthma and allergy admissions occurred prior to the KD illness, suggesting that these allergy/asthma are unlikely to reflect immune dysfunction resulting from KD itself. It may also suggest that a distinct immune phenotype may be associated with an increased risk of both KD and asthma/allergy [19]. In the acute stage of KD, there is sustained neutrophil activation [22], with increased release of human neutrophil elastase and matrix metalloproteinases [23], and similar patterns may be important in childhood asthma [24]. Polymorphisms in the mannose-binding lectin (MBL) gene have been reported to be associated with susceptibility to KD [25]. A role for MBL polymorphisms in susceptibility to asthma and allergic disease has also been reported [26]. This suggests that immunogenetic variations in the innate immune response may contribute to the shared risk of KD and asthma/allergy [19].

In this population-based cohort study, we found that KD patients were 1.52 times more likely than the comparison cohort to develope allergic diseases. These data may reflect the immunological consequences of KD, and/or an underlying susceptibility to both KD and asthma/allergy. The investigation of children following the KD illness does not differentiate between the two possibilities.

The mechanisms by which KD may increase the risk of future development of allergic diseases remain unclear. The immune system is highly activated during KD with a myriad of immunoregulatory changes including Th1 immune related response (such as IFN-gamma, TNF-alpha, IL-1, and IL-10) [27-29] and Th2 immune related response (such as eosinophil, IL-4, IL-5 [30,31]. There is evidence that the scar from prior BCG vaccination and the tuberculin skin test becomes inflated in patients during acute KD and is a marker of Th1-mediated delayed hypersensitivity reaction [32]. Thus, if KD is indeed a strong Th1 trigger and Th1 is associated with a reduced risk of allergic diseases, we would expect that children with KD will have a lower risk of developing allergic diseases. Our data did not provide support for this hygiene hypothesis, where an inverse relationship between increased Th1 cell-mediated inflammation (KD) and decreased Th2-cell mediated allergy is observed [33,34]. By contrast, we observed a positive association between KD and the risk of allergic diseases. The reasons for the differences in the findings are unknown. KD results in an acute inflammation. It has been shown that infections (inflammations) are able to alter the function of regulatory T cells [35]. Our finding of an increased risk of developing allergic diseases following KD may therefore plausibly be related to the effect of regulatory T cells. Clearly, more work will be needed before the influence of immune activation of Th1/Th2 on the risk of developing allergic diseases is understood.

The major strength of our study is the use of a computerized database, which is population based and is highly representative and allows a clear observation of the temporal relationship between KD and allergic diseases. Because we included a national sample of KD patients between 1997 and 2005, and because the control subjects in this study were selected from a simple random sampling of insured general population, we can rule out the possibility of selection bias.

Several limitations of the present study should be noted. First, KD cases were identified using medical diagnoses recorded in the claim database. Data on the accuracy of KD discharge diagnoses is not available in Taiwan. Potential inaccurate data in claims record could also lead to possible misclassification of KD. Given the comprehensiveness of the NHI database and its previous use for incidence study for KD [12], we are confident that the effect of misclassification of case status on our results is most likely of minor importance. Second, diagnoses of allergic diseases rely on administrative claims data may be less accurate than those obtained according to standardized criteria and misclassification is possible. However, this misclassification is likely to be nondifferential and therefore would tend to underestimate rather than overestimate the true relative risk of alleric diseases among KD patients. Third, any study investigating an increased incidence of one disease (allergic disease) conditional on the diagnosis of another disease (KD) in clinical settings may suffer from detection bias. In this study, the fact that KD patients had more physician visit on average than patients in the comparison cohort could make KD patients more likely to have allergies diagnosed. In addition, physicians might be more inclined to find allergic diseases in patients who had KD due to the fact that previous studies have shown the correlation between KD and allergic disease [16-19]. Therefore, the possibility that our findings have arisen from detection bias could not be excluded. Fourth, although we adjusted for several potential confounders in the statistical analysis, a number of possible confounding variables, including body mass index and family history of allergic diseases, which are associated with allergic diseases were not included in our database. Lastly, as with any observational study, residual confounding by unmeasured factors which are different between case cohort and comparison cohort is also possible.

## Conclusion

In conclusion, this study is the first cohort study to investigate epidemiologic data for allergic diseases among children after KD. Our study results provide evidence that KD patients are at an increased risk for the development of allergic diseases compared with the general population.

## Competing interests

The authors declare that they have no competing interests.

## Authors’ contributions

KHC wrote the manuscript. CWC, YKD, YHR and WCL provided essential insight into the interpretation of the results. HSC did the statistical analysis. YCY contributed to study design and interpretation of the data. He had full access to all of the data in the study and took responsibility for the integrity of the data and the accuracy of the data analysis. All authors read and approved the final manuscript.

## Pre-publication history

The pre-publication history for this paper can be accessed here:

http://www.biomedcentral.com/1471-2431/13/38/prepub
